# Constrained flexibility of parental cooperation limits adaptive responses to harsh conditions

**DOI:** 10.1111/evo.14285

**Published:** 2021-06-27

**Authors:** Jeanette B. Moss, Allen J. Moore

**Affiliations:** ^1^ Department of Entomology University of Georgia Athens Georgia 30602 USA; ^2^ Current Address: Department of Evolution, Ecology, and Behavior, School of Integrative Biology University of Illinois at Urbana Champaign Urbana Illinois 61801 USA

**Keywords:** Biparental care, burying beetle, cooperation, environment, plasticity, temperature

## Abstract

Parental care is predicted to evolve to mitigate harsh environments, thus adaptive plasticity of care may be an important response to our climate crisis. In biparental species, fitness costs may be reduced by resolving conflict and enhancing cooperation among partners. We investigated this prediction with the burying beetle, *Nicrophorus orbicollis*, by exposing them to contrasting benign and harsh thermal environments. Despite measurable fitness costs under the harsh environment, sexual conflict persisted in the form of sex‐specific social plasticity. That is, females provided equivalent care with or without males, whereas males with partners deserted earlier and reduced provisioning effort. The interaction of social condition and thermal environment did not explain variation in individual behavior, failing to support a temperature‐mediated shift from conflict to cooperation. Examining selection gradients and splines on cumulative care revealed a likely explanation for these patterns. Contrary to predictions, increased care did not enhance offspring performance under stress. Rather, different components of care were under different selection regimes, with optimization constrained due to lack of coordination between parents. We suggest that the potential for parenting to ameliorate the effects of our climate crisis may depend on the sex‐specific evolutionary drivers of parental care, and that this may be best reflected in components of care.

Parental care is expected to evolve to mitigate hostile and unpredictable environments (Wilson 1975). However, the extent that ecological conditions further modify parenting once it evolves depends on the nature of plasticity of parental care. One potential source of plasticity of care is biparental cooperation. Theoretically, the default role for plasticity in biparental systems is as a mechanism for sexual conflict over which parent cares (Lessells 2012). This can lead to overall care deficits (McNamara et al. 2003; Lessells and McNamara 2012) and, ultimately, to one parent being as effective or more effective at caring for offspring than two parents (Clutton‐Brock 1991; Smiseth et al. 2005; Trumbo 2006). However, the joint rearing of offspring may also allow parents to breed under harsh conditions that would otherwise constrain single parent breeding (Wilson 1975; Emlen 1982). This is because (1) with more than one caregiver, there is more scope for increasing total care allocation (i.e., additive care; Ratnieks 1996; Clutton‐Brock et al. 2001; Johnstone 2011; Savage et al. 2013) and (2) the efforts of a second parent may have synergistic effects on offspring (Pilakouta et al. 2018) and/or offset some costs of care to the primary caregiver (i.e., load lightening; Crick 1992; Johnstone 2011). If true, then transitions to stable biparental care and an increased capacity for cooperation should coincide with expansion into increasingly harsh environments (Wesolowski 1994, 2004).

To date, tests of the “hostile environment” hypothesis as it relates to cooperation over offspring rearing have produced equivocal results (Wynne‐Edwards and Timonin 2007; AlRashidi et al. 2010, 2011; Öberg et al. 2015; Remeš et al. 2015; Wiley and Ridley 2016; Shen et al. 2017; Vincze et al. 2017; Guindre‐Parker and Rubenstein 2018; Lejeune et al. 2019; Lin et al. 2019; Vági et al. 2020). Moreover, the vast majority of insights derive from studies of birds—a group for which biparental care is nearly ubiquitous and rarely decoupled from social monogamy (Cockburn 2006). Consequently, the extent to which suggested links between adverse conditions and enhanced pair coordination (AlRashidi et al. 2010, 2011; Vincze et al. 2017) may be generalizable across taxa is unclear. Transitions to biparental care have occurred repeatedly outside of the avian tree, including in diverse vertebrate (Reynolds et al. 2002) and invertebrate lineages (Trumbo 2012; Suzuki 2013; Gilbert and Manica 2015). Such systems offer rich opportunities to expand the taxonomic scope of investigations into the factors that shape biparental care dynamics.

Burying beetles (Genus: *Nicrophorus*) provide an ideal complement to avian systems for investigating the mechanisms of cooperation and conflict over offspring care (Smiseth 2019), particularly in the context of environmental stress and plasticity. First, burying beetle parental care reflects their ecology. The beetles breed on an ephemeral and widely desirable resource, a dead vertebrate, such that parental care has likely evolved as a strategy to buffer offspring against rapid decomposition and competition (Eggert and Müller 1997; Scott 1998a). Burying beetles are also subsocial; they do not form social associations outside of brief periods of parental care. Therefore, unlike most vertebrates, sources of variation in parental investment can be readily dissociated from other pervasive aspects of social life. Second, we know that there is capacity for plasticity when males and females parent together because biparental males rarely show the same level of effort as uniparental males. Indeed, parental care of burying beetles is sex biased, with females performing the majority of total caregiving duties (Eggert and Müller 1997; Smiseth and Moore 2004; Benowitz and Moore 2016), whereas males provide less direct care in the presence of a female partner (Parker et al. 2015; Pilakouta et al. 2018). Although females may adjust levels of care for variables such as brood size (Smiseth et al. 2007a) and larval maturity (Smiseth et al. 2007b), their quantity or composition of care does not depend on the presence of a male. Conversely, males are highly flexible and capable of adopting larger parental roles as needed to compensate for compromised partner state (e.g., partner loss [Trumbo 1991; Smiseth et al. 2005; Suzuki and Nagano 2009; Parker et al. 2015; Cunningham et al. 2019], handicapping [Creighton et al. 2015], or inbreeding level [Mattey and Smiseth 2015]). Finally, many burying beetles are flexible in the social form of parenting they provide, with uniparental female care, uniparental male care, and biparental care all expressed within natural populations (Trumbo 1991; Smiseth and Moore 2004; Suzuki and Nagano 2009; Benowitz et al. 2016; Scott 1998a). If it is true that multiple parents provide more effective care to offspring in hostile conditions (i.e., through additive and/or load‐lightening effects), then members of the more flexible sex should also be less inclined to withhold care in response to a generalized environmental stressor, which may compromise the states of both parents.

Here, we use *Nicrophorus orbicollis*—a primarily biparental species and among the few members of the temperate species complex to have successfully expanded into the warmer climate of the U.S. southeast (Trumbo 1990)—to examine the role that plasticity of parental investment plays in mitigating harsh ambient conditions. High temperatures, as occur at low latitudes, are generally implicated in more costly and less profitable reproduction in burying beetles (Meierhofer et al. 1999; Müller et al. 2007; Steiger et al. 2007; Jacques et al. 2009; Quinby 2016; Ong 2019; Feldman 2020). Individuals breeding under these conditions have been found to suffer reduced lifespans and lower lifetime reproductive success (Laidlaw 2015). We used a mixed factorial design with repeated measures to test whether beetles acclimated to high‐temperature (i.e., harsh) breeding conditions are capable of mitigating effects through adjustments in parental care. We quantified within‐subject behavioral comparisons to examine the extent that sex‐specific plasticity and the capacity for biparental care drive responses to hostile environments. Our prediction was that if conflict associated with social plasticity leads to overall deficits in care, and increased care is key to mitigating environmental stress, then an adaptive response should be reflected in a significant interaction between social condition and thermal environment. On the contrary, we found that sexual conflict persisted even in the face of higher fitness costs associated with care. Using standardized selection gradients, we show that failure of parents to increase cooperation over care can likely be explained by stabilizing selection on care at higher temperatures, with additive contributions of care generally correlating with reduced breeding performance. This occurs because the components of care are under different forms of selection, the components are not independent, and individual variation did not reflect a plastic response to subtle variation in their partner's behavior.

## Methods

### STUDY SYSTEM

*Nicrophorus orbicollis* is a large‐bodied, ecological generalist that breeds on small (∼20 g) to medium (∼100 g) vertebrate carcasses in North American woodlands. The species has a large latitudinal distribution (from southern Canada to northern Texas), with breeding seasons at the southern margins characterized by higher daily temperature extremes (3−8°C on average) and a greater frequency of reproductive failure (Trumbo 1990). As with most members of the genus, parental care is elaborate and extends into the posthatching stage (Eggert and Müller 1997; Scott 1998a). During prehatching stages, parents work together to bury and prepare the carcass by removing hair and applying anal secretions to prevent microbial growth. During the posthatching stage, parents continue to maintain the brood ball and directly provision to begging young via regurgitation. Although larvae of most burying beetles can survive without parents (Schrader et al. 2015; Jarrett et al. 2018), *N. orbicollis* show obligate parental care, meaning that larvae depend on direct provisioning for survival (Trumbo 1992; Capodeanu‐Nägler et al. 2016, 2018). Parental care is described as predominantly biparental on the basis that males and females typically overlap with each other in the posthatching stage (in 66% of cases; Benowitz and Moore 2016), and both sexes perform the full repertoire of parenting behaviors (Scott and Traniello 1990; Trumbo 1991; Scott 1998a). However, as is the case with any reproductive systems studied in detail, individual investment is highly flexible and subject to environmental and social pressures (Trumbo 1991; Scott 1998b; Creighton et al. 2015).

### FIELD COLLECTION AND HUSBANDRY

*Nicrophorus orbicollis* were captured from Whitehall Forest, Athens GA, in the summer of 2020. Beetles were baited into hanging traps with salmon and collected twice weekly to breed an outbred laboratory colony. Simultaneously, Thermochron^®^ iButton temperature loggers (©Maxim Integrated Products, Inc., San Jose, CA) were deployed ∼10−12 cm underground at trap locations throughout our collection site to estimate the range of temperatures beetles likely experience in their subterranean brood chambers. *Nicrophorus orbicollis* begin emerging from hibernation in early spring and reach peak densities around midsummer (between late June and early August; Ulyshen and Hanula 2004). In 2020, mean daily temperatures during these two potential breeding windows—late spring/early summer (31 May to 03 July) and mid/late summer (15 July to 22 August)—ranged between 21.71 ± 1.38°C and 23.82 ± 0.77°C, respectively (Fig. S1). Diurnal temperature fluctuations were between 0.75 and 7.71°C. To capture this variation in the laboratory, we programed two incubators to ramp between set points of diurnal temperatures over the course of 10:14 hour reverse light:dark cycles, simulating early and late summer breeding conditions, respectively. The first treatment, hereafter the “benign” thermal environment, was set to ramp between 19°C (night) and 20°C (day), whereas the second, hereafter the “harsh” thermal environment, was set to ramp between 23°C (night) and 24°C (day). Focal individuals for the experiment were selected from the F01 and F02 generations of laboratory‐bred beetles (bred on countertops at room temperature [20 ± 0.5°C]). Individuals were divided evenly between the treatment incubators on the third day of pupal development to facilitate acclimation (adults eclosed into the environment in which they would ultimately breed) while controlling for possible early developmental effects of temperature. All virgins selected for the experiment were at least 14 days of age.

### BREEDING TRIALS

**Figure 1:**
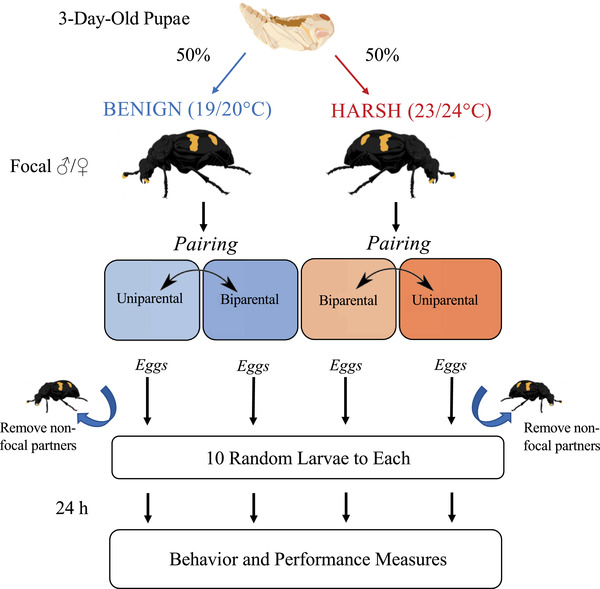
Schematic of mixed factorial experimental design. Focal individuals of each sex were divided evenly between the treatment incubators on the third day of pupal development to facilitate thermal acclimation (thermal environment = between‐subject factor). At pairing, individuals were assigned randomly to a starting social condition (uniparental or biparental) and restarted in the opposite social condition upon successful completion of a first trial (social condition = within‐subject factor). In uniparental trials, nonfocal parents were removed after egg laying. All eggs were collected prior to hatching and each widowed parent or biparental pair was allocated a standardized number of larvae (*N* = 10) of random genetic origin. Behavioral and performance measures were collected starting 24 h into care.

Breeding trials were carried out between October 2020 and January 2021. We used a mixed factorial design as outlined in Figure 1, in which social condition (uniparental or biparental) was measured as a within‐subject factor and thermal environment (Benign or Harsh) was measured as a between‐subject factor. The goal was to achieve a balanced experimental design with respect to the number of individuals undergoing repeated trials (*N* = 20 males and females per thermal environment), which would allow us to explicitly quantify differences in individual plasticity between the two thermal environments. To facilitate this, we randomized the order in which focal individuals were exposed to either social condition. To create the biparental condition, individuals were paired to a focal individual of the opposite sex within the same thermal environment. To create the uniparental condition, individuals were paired to a random unrelated beetle of the opposite sex (also within the same thermal environment) that would be removed between egg laying and hatching. Individuals that successfully completed their first trial would continue on to a second trial in the opposite social condition, whereas individuals that failed their first trial within 7 days of pairing were restarted. To account for higher breeding failure among virgins (primarily due to eggs being unfertilized; Table S1), beetles were allowed one failure on their first attempt.

At the start of each trial, pairs were placed in a plastic box (17.2 × 12.7 × 6.4 cm; Pioneer Plastics, Dixon, KY) filled with approximately 2 cm of moistened potting soil and containing a freshly thawed mouse carcass between 40 and 45 g (RodentPro, Evansville, IN). Boxes were returned to the incubator where they were kept on a darkened shelf beneath blackout curtains to simulate an underground breeding environment. From pairing, breeding boxes were checked twice daily for eggs. Pairs with no eggs after 7 days were restarted on a new mouse. Two days after eggs were first recorded, the brood ball and focal beetle(s) were transferred to a new breeding box (nonfocal parents were removed) such that eggs could be collected and counted. This step was performed to facilitate brood standardization, which ensured that comparisons of performance would be attributed to parental care rather than differences in fertility or genetic quality. Eggs were placed in petri dishes with damp filter paper and monitored every 8 h until larvae appeared. At this stage, synchronously hatching broods were randomly mixed, and each pair of fertile parents was given exactly 10 larvae. Broods that failed to hatch within 5 days of laying were recorded as unfertilized, and the pair was restarted.

### BEHAVIOR AND PERFORMANCE MEASURES

Behavioral observations were carried out 24 h after introducing larvae, as previous work indicates that offspring provisioning peaks around this time (Smiseth et al. 2003). Breeding boxes were placed in a dark, temperature‐controlled observation room (20°C) and allowed to acclimate for 30 min, ensuring that observed differences in parenting could not be attributed solely to temperature‐dependent activity. Observations took place under red light over a 30‐min period. Behaviors were recorded every minute via instantaneous scan sampling. These included any instances of direct provisioning (i.e., mouth‐to‐mouth contact suggesting regurgitation of food to larvae), self‐feeding (i.e., opening up the cavity with mouth or consuming carcass to facilitate subsequent regurgitations), offspring association (i.e., in physical contact with larvae but not provisioning), carrion maintenance (i.e., positioned under brood ball or walking over brood ball exuding antimicrobial secretions), on carcass not attending (i.e., grooming or simply not providing care), and off brood ball. Behaviors were treated as mutually exclusive and only one was recorded at each time interval; however, specific behaviors were often difficult to functionally disentangle. For example, bouts of direct provisioning were often interrupted with brief periods of offspring association, and “self‐feeding” was almost always followed by direct provisioning of larvae (pers. obs.). To enable the most meaningful interpretation of these data, three originally distinct behaviors—direct provisioning, self‐feeding, and offspring association—were collapsed into a more general category, Direct Care. “Carrion maintenance” was classified as Indirect Care, and “not attending” and “off brood ball” were classified as No Care. This gave each individual three scores, which together summed to 30.

After completing observations, brood boxes were returned to incubators and subsequently checked three times per day for parental desertion. Desertion was inferred when beetles were observed buried in the dirt away from the brood ball for three consecutive observations (Hopwood et al. 2015; Parker et al. 2015; Benowitz and Moore 2016). At this point, beetles were removed, and we recorded the duration of care (in days). Final weights were taken for each beetle at the end of a breeding trial, and those due for a second trial were fed and returned to the incubator for 1−2 days prior to restarting. To calculate and compare performance across trials, we measured two traits implicated in parental performance: total number of offspring surviving to the end of a breeding trial and mean larval mass (Parker et al. 2015). These measures were taken only after larvae dispersed naturally from the brood ball, as to ensure maximal feeding time.

### STATISTICAL ANALYSES

All statistical analyses were conducted in R version 4.0.3 (R Core Development Team 2019) using the package lme4 (Version 1.1‐26; Bates et al. 2015). We first examined evidence for fitness costs associated with the high temperature environment, beginning with basic life history parameters. Because a large number of adult deaths were recorded over the course of our experiment, our first analysis was of parental longevity. We used a Cox proportional hazard regression model implemented in the R package “survival” (Version 0.5.5; Therneau and Lumley 2015) to test the association between thermal environment and mortality across all attempted trials, adjusting for sex. We further tested for differences in reproductive parameters attributable to temperature: specifically, we used simple linear regression to estimate effects on fecundity (number of eggs laid) and fertility (egg hatching success) across all trials. We included female body size (measured as pronotum width, in mm) as a covariate in these models, and found significant effects of female size on fecundity (df = 1, 266, *F* = 26.264, *P* < 0.001) but not fertility (df = 1, 251, *F* = 0.249, *P* = 0.618). We next compared the breeding outcomes between thermal environments following brood standardization. Retaining only beetles that completed at least one trial, we estimated the magnitude of effect of temperature on development time (days between introducing larvae to the brood ball and larval dispersal), number of larvae dispersing, and mean larval mass. Because the maximum brood size under our manipulation was 10, larval number was fit to a generalized linear model (glm) with a binomial distribution as to estimate proportional “successes.” We fit development time to a glm with a Gamma distribution, and larval mass to a simple linear model. These effects were estimated independently of specific parental behaviors; however, initial models considered breeding history (binary specifying at least one previous breeding success between parents) and carcass size as covariates to account for possible variation arising from parenting experience or resource volume. Neither variable had significant effects on development time (df = 1, 155; breeding history: *t* = 0.835, *P* = 0.405; carcass size: *t* = −0.761, *P* = 0.448) or larval mass (df = 1, 155; breeding history: *F* = 0.006, *P* = 0.939; carcass size: *F* = 1.420, *P* = 0.235), and so were removed. However, carcass size had significant effects on the number of larvae dispersed (df = 1, 161; breeding history: *z* = 1.299, *P* = 0.194; carcass size: *z* = −2.954, *P* = 0.003), and so this variable was retained in the final model.

After identifying costs associated with thermal stress, we evaluated evidence for variation in parenting behaviors, specifically as it pertained to cooperation between males and females. We asked whether social plasticity—a strategy that typically leads to sexual conflict over care via reduced relative contributions of one partner—is relaxed in pairs coping with high environmental stress, indicating more equal investment between uniparental and biparental conditions. First, we characterized plasticity of males and females within environments, predicting that individual parental effort should decline between uniparental and biparental conditions if biparental care defaults to sexual conflict. To test this, we retained only individuals with repeated measures of behavior and performed repeated‐measures ANOVAs for each sex and thermal environment separately, analogous to estimating reaction norms. Social condition was specified as the within‐subject factor and individual ID nested within trial number was the error term. Second, we compared plasticity between environments, predicting that the slope of the change in parental effort between social conditions should be shallower in the harsh environment if social plasticity (i.e., underpinning sexual conflict) is relaxed. This was tested using a repeated‐measures multivariate ANOVA (RM MANOVA) in the R package, MANOVA.RM (Version 0.4.3; Friedrich et al. 2019). The three behavioral metrics—duration of care, time in Indirect Care, and time in Direct Care—were specified as response variables and thermal environment was specified as a between‐subject factor. Attendance times were divided by the mean temperature‐dependent development times to facilitate comparison between thermal conditions. Wald‐type statistics (WTS) and resampling *P‐*values are reported for within‐ and between‐subject factors and their interaction.

Finally, to better understand the impetus (or lack thereof) for variation in parental plasticity, we examined how selection on *cumulative* parenting effort differed between thermal environments. To achieve this, we leveraged all successful trials from either environment and calculated the cumulative care experienced by offspring in each. Hence, uniparental males and females could provide up to 30 units of care, whereas biparental pairs could provide up to 60. To gain an initial impression of how variation in care affected offspring, we visualized a standardized offspring performance trait (larval mass) as a function of cumulative parental time spent in any type of care (Direct or Indirect, summing to Total Care) and inspected the shape of the curve. To formally quantify and compare selection acting on individual components of care, we then calculated standardized selection gradients following Lande and Arnold (1983) and Brodie et al. (1995). Briefly, fitness measures (number of larvae and mean larval mass) were regressed on overall parenting experienced by larvae (maximum number of days attended, Cumulative Direct Care, and Cumulative Indirect Care) and both linear and nonlinear components of selection were estimated. We then examined intra‐environmental variation in parenting as a function of social condition by performing a mixed model analysis of variance (ANOVA) for each metric followed by specified a priori pairwise contrasts, comparing uniparental and biparental care behavior within a sex. Contrasts were implemented using the lsmeans package (Version 2.30‐0; Lenth 2016). To control for repeated measures of focal parents, we included male and female IDs as random block effects in the model design.

Full data are available from Dryad (Moss and Moore 2021).

## Results

Over the course of this experiment, we initiated 358 breeding trials spread over two thermal environments and three social conditions. Only 165 trials resulted in larvae that survived through the 24‐hr behavioral observation period (see Table S1 for sources of failure). These included 32 biparental pairs, 29 uniparental males, and 30 uniparental females in the harsh environment, and 26 biparental pairs, 23 uniparental males, and 25 uniparental females in the benign environment. The final number of focal individuals with repeated measures amounted to 18 males and 20 females in the harsh environment and 20 males and 20 females in the benign environment.

### FITNESS COSTS OF THERMAL STRESS

**Figure 2:**
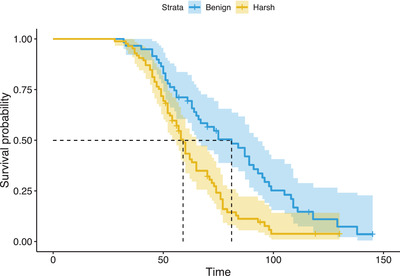
Survival curves calibrated from the mortality times of 144 beetles (25 censored) assigned to the benign (blue line; *N* = 59) and harsh (orange line; *N* = 85) thermal environments. Dotted lines indicate median lifespans for each environment, in days.

**Figure 3:**
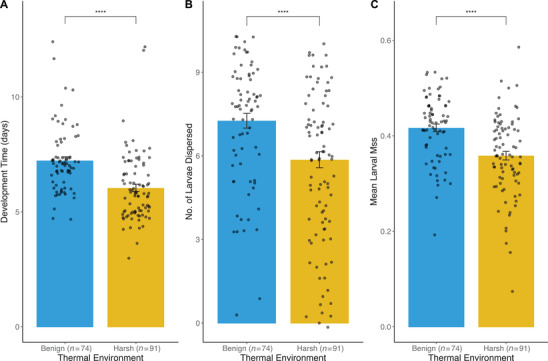
Comparisons of breeding performance across all social conditions between the benign and harsh thermal environment. Performance is compared based on (A) number of days between larval introduction and dispersal, (B) number of larvae dispersed, and (C) mean larval mass (in g). Raw data points are overlain on boxplots to illustrate the distribution of performance measures. Asterisks indicate significant differences at *P* = 0.0001.

We observed strong adverse effects on fitness associated with thermal stress. Focal beetles in the harsh environment suffered a 47.6% higher mortality risk compared to counterparts in the benign environment (95% CI [1.43, 3.09], *P* < 0.001; Fig. 2), with males outliving females (HR = 0.62, 95% CI [0.43, 0.90], *P* = 0.013). Within breeding trials, mortality accounted for 21.7% of failures among inexperienced breeders, and 31.6% among experienced breeders (compared to 12.9% and 10.2% in the benign environment, respectively). Reproductive life history parameters were also affected by temperature, with beetles laying fewer (df = 1, 266, *F* = 8.324, *P* = 0.004) and less fertile (df = 1, 251, *F* = 4.691, *P* = 0.031) eggs under thermal stress prior to brood standardization. In trials that progressed through the dispersal stage (post‐brood standardization), larvae developed significantly faster in the harsh environment compared to the benign environment (df = 1, 157, *t* = 5.104, *P* < 0.001; Fig. 3A) and were reduced for both number (df = 1, 163, *z* = −6.982, *P* < 0.001; Fig. 3B) and mass (df = 1, 157, *F* = 22.487, *P* < 0.001; Fig. 3C).

### PLASTICITY OF BIPARENTAL CARE

**Figure 4:**
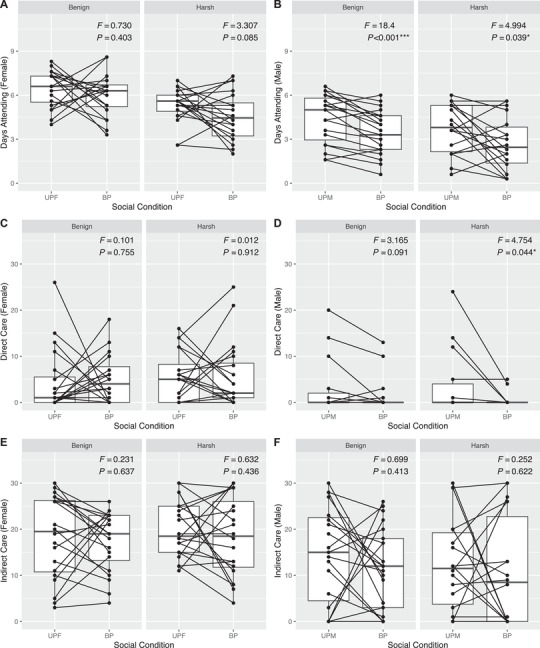
Reaction norm‐type plots depicting social plasticity in components of parental care within thermal environments. Individual data points overlain on boxplots representing the sample distribution under uniparental (UPF = uniparental female; UPM = uniparental male) and biparental (BP) care. Lines connecting points represent within‐individual plasticity, where negative slopes illustrate reductions in care between uniparental and biparental conditions. Panels separate benign and harsh thermal environments. These trends are depicted for (A) female attendance time, (B) male attendance time, (C) female Direct Care, (D) male Direct Care, (E) female Indirect Care, and (F) male Indirect Care. Test statistics were obtained from repeated measures ANOVA tests with social condition (uniparental or biparental) as the within‐subject factor and individual ID nested within trial number as the error term.

Despite high fitness costs, we found no evidence for increased cooperation between parents in the harsh environment. This was caused by the fact that individuals adjusted their behaviors for social condition, but plasticity itself did not adjust for harsher environmental conditions. Examining these patterns within thermal environments, it was clear that plasticity was sex‐specific and accounted for significant variation in parental effort independent of temperature. Although females were not plastic and provided the same level of care regardless of male presence or absence (Fig. 4A, C, E), males deserted the brood significantly earlier in the presence of a female compared to when caring alone (Fig. 4B). These trends held in separate analyses of each thermal environment. Further, biparental males were seen to provision less than uniparental males in the harsh environment, but not in the benign environment (Fig. 4D). Neither sex in either environment adjusted levels of indirect care in response to social condition (Fig. 4E, F). Comparing plasticity between thermal environments largely recapitulated sex‐specific patterns, with social condition emerging as a significant predictor of overall within‐subject behavioral variation in males (WTS = 15.691, *P* < 0.001) but not in females (WTS = 4.816, *P* = 0.185). However, neither males nor females showed significant differences in parenting behavior between thermal environments (Males: WTS = 0.399, *P* = 0.945; Females: WTS = 3.752, *P* = 0.295). Moreover, the interaction of within‐treatment (social condition) and between‐treatment (thermal environment) effects was not significant, failing to support the patterns we predicted for an adaptive shift from conflict to cooperation.

### SELECTION ON CARE

**Figure 5:**
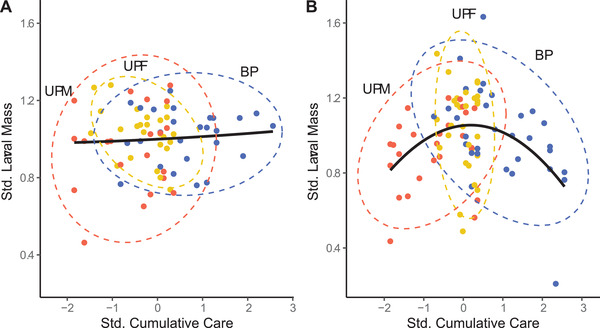
Relationship between standardized total parental time allocation to care (direct + indirect) and standardized mean larval mass in the (A) benign environment, and (B) harsh environment. Data points belonging to each of three social conditions (uniparental male [UPM], uniparental female [UPF], and biparental [BP]) are differentiated by color (red = UPM, yellow = UPF, blue = BP). Labeled ellipses illustrate 95% confidence intervals, and approximate splines illustrate overall trends in the data.

To understand why biparental pairs exposed to a generalized stressor did not cooperate to increase overall care, we inspected the relationship between cumulative care and a larval performance trait (mean larval mass). We found that although larvae raised by two parents typically experienced more care overall than larvae raised by one parent, additive contributions beyond the maximum achievable under uniparental care appeared to provide negligible fitness benefits under benign conditions (Fig. 5A) and became harmful under stressful conditions (Fig. 5B). To identify which parental behaviors were driving these patterns, we calculated selection gradients acting on individual components of care. Duration of parental attendance had significant linear effects on the number of offspring reared to dispersal in both environments (Table 1A, B). However, selection acting on Direct versus Indirect Care differed between the two thermal environments. Although in the benign environment there was no statistically significant selection acting on either component, in the harsh environment we found significant directional and stabilizing selection for different care components. Although brood size showed a positive linear relationship with Cumulative Direct Care, extreme values of Cumulative Indirect Care had significant nonlinear effects on both larval size and number. Thus, the harsh environment selected for increased time in direct provisioning and intermediate time in carcass maintenance. Variation was largely attributed to the number and sex of parents, as all behaviors varied significantly with social condition under both environmental conditions (Table 2A, B) and pairwise comparisons identified only Indirect Care as being significantly increased for biparental pairs relative to uniparental females (Table 3).

**Table 1:** Linear (*β*) and nonlinear (*γ*) standardized selection gradients (Lande and Arnold 1983; Brodie et al. 1995) relating brood performance (measured as number of larvae dispersed and mean larval mass [in g]) to behavioral measures of parenting effort. Selection gradients are presented separately for the (a) benign and (b) harsh environment. Statistically significant gradients (at *α* = 0.05) are highlighted in bold

	Larval number						Mean larval mass			
Behavioral measure	*N*	*β*	*P*	*γ*	*P*	*N*	*β*	*P*	*γ*	*P*
***(a) Benign environment***										
Days attended	74	**0.124** (±0.034)	<0.001	–0.259 (±0.078)	0.078	73	0.028 (±0.021)	0.179	–0.006 (±0.050)	0.907
Total Indirect Care	74	0.058 (±0.035)	0.097	–0.025 (±0.052)	0.632	73	–0.004 (±0.020)	0.832	–0.005 (±0.032)	0.867
Total Direct Care	74	0.042 (±0.034)	0.228	0.008 (±0.050)	0.868	73	0.028 (±0.019)	0.164	–0.021 (±0.031)	0.513
***(b) Harsh environment***										
Days attended	91	**0.161** (±0.052)	0.002	–0.089 (±0.131)	0.497	86	0.039 (±0.027)	0.167	0.035 (±0.068)	0.610
Total Indirect Care	90	–0.001 (±0.052)	0.992	**–0.165** (±0.069)	0.020	85	–0.037 (±0.027)	0.179	**–0.100** (±0.035)	0.006
Total Direct Care	90	**0.102** (±0.051)	0.049	0.008 (±0.060)	0.896	85	0.021 (±0.026)	0.416	–0.053 (±0.029)	0.069

**Table 2:** Mixed model ANOVAs testing the effects of social condition (uniparental male, uniparental female, and biparental) on parental effort (the total time allocated to indirect or direct care during a 30‐min observation) for three forms of care. Results are reported separately for the benign and harsh environment, with male and female IDs treated as random factors

Model	MS	Num *df*	Den *df*	*F*	*P*
***(a) Benign environment***					
Days attended	20.06	2	71.00	**8.60**	<0.001
Total Indirect Care	5.00	2	37.75	**12.34**	<0.001
Total Direct Care	96.68	2	40.05	**6.22**	0.004
***(b) Harsh environment***					
Days attended	14.37	2	57.81	**15.65**	<0.001
Total Indirect Care	18.05	2	61.10	**27.36**	<0.001
Total Direct Care	124.98	2	63.29	**3.20**	0.047

**Table 3:** A priori determined pairwise comparisons of parental effort among pairs within the same acclimation environment, benign or harsh. For each dataset, biparental (BP) is the reference group against which uniparental female (UPF) and uniparental male (UPM) observations are contrasted. Effects with statistically significant *P‐*values (at *α* = 0.05) are shown in bold

*Contrast*		*t*	*P*
***(a) Benign environment***			
Days attended	BP—UPF	–0.836	0.684
	**BP—UPM**	3.080	0.010
Total Indirect Care	**BP—UPF**	3.885	0.001
	**BP—UPM**	4.119	<0.001
Total Direct Care	BP—UPF	1.250	0.430
	**BP—UPM**	3.408	0.005
***(b) Harsh environment***			
Days attended	BP—UPF	0.069	0.997
	**BP—UPM**	4.967	<0.001
Total Indirect Care	**BP—UPF**	3.952	0.006
	**BP—UPM**	7.127	<0.001
Total Direct Care	BP—UPF	0.391	0.919
	BP—UPM	2.328	0.064

## Discussion

In this study, we investigated the potential of plasticity of biparental care to ameliorate the effects of a harsh environment in a burying beetle, *N. orbicollis*. Our prediction was that offspring receiving more care through additive or load lightening benefits of multiple caregivers would fare better under harsh environmental conditions, and therefore strategies that promote sexual conflict between parents should be relaxed in favor of strategies that promote cooperation. We tested this by exposing families with different parental compositions to thermal stress and characterizing individual social plasticity (i.e., adjustments in care in response to partner presence versus absence) in both benign and harsh environments. Our data support that investment decisions are sex specific and sensitive to social condition; however, these patterns were not affected by generalized stress on the family. Irrespective of thermal environment, females were unresponsive to male partners and males with partners withheld contributions of direct care. To explain this, we used standardized selection gradients to quantify the importance of cumulative parental behaviors for predicting environment‐dependent offspring performance. Contrary to our predictions, the harsh environment favored intermediate, not high overall parental investment. Moreover, we found that the type of care was important, and components were not independent of each other. These results challenge our understanding of the adaptive role of biparental care in hostile environments.

The thermal stress we imposed had strong deleterious fitness effects compared to a more benign temperature. Not only did adults acclimated to the warmer environment suffer reduced life spans and lower reproductive potential per bout, but offspring were also less likely to survive to dispersal and attained lower body mass than counterparts in the benign environment. Our results are consistent with both field and laboratory studies of the genus noting significant performance declines along gradients of temperatures (Meierhofer et al. 1999; Müller et al. 2007; Steiger et al. 2007; Jacques et al. 2009; Quinby 2016; Ong 2019; Feldman 2020). Given these severe fitness costs, we expected there would be selection pressure to cope with extreme temperatures.

Our expectation was that because burying beetles show flexibility in parenting in response to social parameters (Trumbo 1991; Smiseth et al. 2005; Suzuki and Nagano 2009; Creighton et al. 2015; Mattey and Smiseth 2015; Parker et al. 2015), and males caring with females have “spare capacity,” the application of a generalized stressor should promote shifts from conflict to cooperation. Instead, we found that both males and females adhered to predicted sex‐specific rules for parental investment—males were plastic and females were not, as seen in the related *N. vespilloides* (Smiseth et al. 2005; Royle et al. 2014)—irrespective of the selective environment. Specifically, females cared at capacity even when exposed to heat stress and provided with male helpers, allowing us to reject any “load lightening” benefits of two caregivers (Crick 1992; Johnstone 2011). Males in the presence of females withheld direct care and deserted broods earlier, consistent with sexual conflict. Moreover, we failed to detect any significant interaction between social condition and thermal environment, suggesting that the social plasticity underpinning conflict is not relaxed under stressful conditions.

To understand the lack of cooperation in biparental pairs exposed to a harsh environment, it is necessary to examine selection acting on cumulative investment. Because environmental hostility exacerbates offspring vulnerabilities, we expected that *N. orbicollis*—a species with dependent young—would benefit from the capacity to increase parental investment when confronted with more extreme environments (Wilson 1975; Wesolowski 1994, 2004). Contrary to this expectation, our high‐stress environment did not induce strong and consistent directional selection relative to the benign environment. Instead, we found that increased overall care was associated with significant nonlinear effects—an indication of strong stabilizing selection (Schluter 1988). This translated to fewer and smaller offspring among caregivers with both the lowest and the highest cumulative behavioral investments (Fig. 5B). We detected no improvements in performance among families with two caregivers as opposed to one (Fig. 5B). In fact, because two caregivers are effectively capable of twice the total effort, biparental pairs accounted for much of the performance reduction in the upper tails of the care distribution. Our results are consistent with independent investigations carried out in Oregon (Feldman 2020) and Canada (Ong 2019), which report significantly reduced performance and limited compensation among biparental pairs exposed to experimental warming treatments. Our study provides a mechanism for these effects: reduced offspring performance at higher temperatures does not result from biparental care per se, but from temperature‐dependent thresholds in optimal care allocation, which are most likely to be exceeded when two parents are active at the nest.

It is important to note that the selection gradients that emerged under harsh conditions differed depending on the specific component of care considered. Although both duration of care and time in direct care were under positive directional selection, the major driver of stabilizing selection was time in indirect care (Table 1). Indeed, the main difference between biparental pairs and uniparental females across environments was an increase in indirect care, underpinned by the fact that neither males nor females showed plasticity of this behavior. A possible explanation for why offspring fitness appeared to decline with increased cumulative indirect care is that saturation of one care type necessitates trade‐offs with others. Indeed, negative genetic correlations between indirect care and direct care have been described in quantitative genetic work in the related *N. vespilloides* (Walling et al. 2008) and could limit the ability of one or both parents to optimize time budgets for a particular environment. As seen above, females tend to maximize their time in care and are unresponsive to males, such that females with high time budgets for indirect care may be constrained from reallocating time to other behaviors (i.e., due to a high perceived cost of reducing time in indirect care). Meanwhile, males are unlikely to be a source of additive direct care due to the sex‐specific nature of their plasticity (i.e., they withhold provisioning effort when females are present and do not adjust for environmental stress). As a result of these individually maladaptive responses to stressful environments, two parents are not more efficient at caring for offspring than one.

Given the predominance of biparental care in *N. orbicollis*, why have mechanisms for enhancing coordination not evolved? In systems where brood care responsibilities are shared by more than one individual, social factors are expected to have an outsized influence on investment decisions, and the ability to mount coordinated responses may help buffer environmental variation (Heinsohn 2004; Ridley and Raihani 2008). Models of biparental care such as partial compensation (Houston and Davies 1985), negotiation (McNamara et al. 1999), and turn‐taking (Johnstone et al. 2014) assume that male and female strategies are optimized to resolve conflict over offspring care. However, if biparental care of burying beetles did not evolve to mitigate offspring need, then the dynamics predicted under these models may not hold true. Parental care in burying beetles is elaborate, requiring investment not only during posthatching care of offspring but also during prehatching resource defense and preparation. In a hot and stressful environment, a pairs’ ability to bury a carcass efficiently and to great depths may have an outsized influence on breeding outcomes. Hence, the transition to biparental care could have facilitated the colonization of harsh environments via cooperation over other aspects of care, which were not measured here. A further explanation for the observed lack of male response to offspring need is that sexual conflict continues to structure interactions between the sexes, as in the related *N. vespilloides* (Boncoraglio and Kilner 2012; Parker et al. 2015). Although evidence from a variety of burying beetle species suggests that the compensatory role of males during posthatching care is fully observable in the case of partner removal (Smiseth et al. 2005; Suzuki and Nagano 2009; Royle et al. 2014), more subtle perturbations in the family environment, such as reduced partner provisioning (Suzuki and Nagano 2009; Suzuki 2020), increased offspring begging (Suzuki 2020), or, as we show here, the application of a generalized stressor, often fail to induce compensation. Thus, our results are consistent with males adopting an “insurance policy” strategy for participation in care, remaining impervious to offspring needs except in the extreme case that the female dies or abandons the nest (Parker et al. 2015). Overall, research on burying beetles suggests that there can be sex‐specific evolutionary pathways for biparental care consistent with sexual conflict as one of the drivers of the evolution of care in this genus (Boncoraglio and Kilner 2012; Parker et al. 2015).

The prediction that cooperative parental strategies enhance resilience in harsh or hostile environments is not novel (Wilson 1975; Emlen 1982), but climate change has afforded new urgency to understanding its practical significance (Lucey et al. 2015; Manfredini et al. 2019; Henriques and Osmond 2020). Our study has shown that burying beetles at southern range margins will face steep reproductive challenges associated with rising temperatures alone, and that these will not be alleviated through biparental cooperation. Despite the predominance of biparental social structures in this species, strategies for coordinated care are unrefined. The implication of our work is that the potential for parenting to ameliorate the effects of climate change is likely to depend on the evolutionary drivers of parental care, which may be sex specific and be best reflected in components of care.

## AUTHOR CONTRIBUTIONS

JBM and AJM conceived and designed the study. JBM collected the data and performed analyses. JBM and AJM wrote this article. Both authors gave final approval for publication.

## CONFLICT OF INTEREST

The authors declare no conflict of interest.

## DATA ARCHIVING

All data available in Dryad: doi.org/10.5061/dryad.59zw3r26s.

Associate Editor: C.Linnen

Handling Editor: T. Chapman

## Supporting information



Supplementary material
