# Supplementary material for: Constrained flexibility of parental cooperation limits adaptive responses to harsh conditions
Source: Evolution. 2021 Jun 27;75(7):1835–49. doi: 10.1111/evo.14285 (PMC8362138; doi:10.1111/evo.14285)
Supplement: Supplementary file 1 — Supplementary material [file EVO-75-1835-s001.docx]

**Figure S1:** Trends in ambient temperature in Whitehall Forest, Athens, GA measured over two periods of the active season of *Nicrophorus orbicollis*: between 31 May–03 July 2020 (‘Early Summer’; top) and 15 July–22 August (‘Late Summer’; bottom). Temperature data were collected every 30 minutes using Thermochron® iButton temperature loggers (©Maxim Integrated Products, Inc., San Jose, CA, U.S.A) deployed 10-12 cm underground at beetle trap locations. This was done to ensure that the microenvironments where burying beetles are most likely to breed (underground, beneath the forest canopy) were represented. In each panel, points represent temperature recordings, where daytime temperatures (0700-2000) are shown in orange and nighttime temperatures (2000-0700) are shown in blue. Lines represent trends in daily mean temperature.

**Table S1:** Sources of breeding failure across initial and subsequent breeding attempts. First breeding attempts include those in which neither focal parent had a previous breeding success. Subsequent breeding attempts include those in which one or both focal parents had previous breeding successes. Sources of breeding failure include mortality of one or both parents, absence of eggs after seven days, presence of eggs within seven days but absence of fertilization, and total cannibalization. Rates are reported as raw numbers and percentages.

| **Source of Failure** | **First Breeding Attempt**  **(breeding experience = 0)** | | **Subsequent Breeding Attempts**  **(breeding experience = 0)** | |
| --- | --- | --- | --- | --- |
|  | **Benign (n=77)** | **Harsh (n=124)** | **Benign (n=59)** | **Harsh (n=98)** |
| Mortality | 10 (13.0%) | 27 (21.8%) | 6 (10.2%) | 31 (31.6%) |
| No eggs | 8 (10.4%) | 13 (10.5%) | 7 (11.9%) | 10 (10.2%) |
| Eggs but not fertilized | 12 (15.6%) | 19 (15.3%) | 2 (3.3%) | 5 (5.1%) |
| All larvae cannibalized | 10 (13.0%) | 13 (10.5%) | 7 (11.9%) | 12 (12.3%) |
